# Soybean Knowledge Base (SoyKB): a web resource for soybean translational genomics

**DOI:** 10.1186/1471-2164-13-S1-S15

**Published:** 2012-01-17

**Authors:** Trupti Joshi, Kapil Patil, Michael R Fitzpatrick, Levi D Franklin, Qiuming Yao, Jeffrey R Cook, Zheng Wang, Marc Libault, Laurent Brechenmacher, Babu Valliyodan, Xiaolei Wu, Jianlin Cheng, Gary Stacey, Henry T Nguyen, Dong Xu

**Affiliations:** 1 Department of Computer Science, University of Missouri, Columbia, MO 65211, USA; 2 Christopher S. Bond Life Sciences Center, University of Missouri, Columbia, MO 65211, USA; 3 National Center for Soybean Biotechnology, University of Missouri, Columbia, MO 65211, USA; 4 Informatics Institute, University of Missouri, Columbia, MO 65211, USA; 5 Division of Plant Sciences, University of Missouri, Columbia, MO 65211, USA

## Abstract

**Background:**

Soybean Knowledge Base (SoyKB) is a comprehensive all-inclusive web resource for soybean translational genomics. SoyKB is designed to handle the management and integration of soybean genomics, transcriptomics, proteomics and metabolomics data along with annotation of gene function and biological pathway. It contains information on four entities, namely genes, microRNAs, metabolites and single nucleotide polymorphisms (SNPs).

**Methods:**

SoyKB has many useful tools such as Affymetrix probe ID search, gene family search, multiple gene/metabolite search supporting co-expression analysis, and protein 3D structure viewer as well as download and upload capacity for experimental data and annotations. It has four tiers of registration, which control different levels of access to public and private data. It allows users of certain levels to share their expertise by adding comments to the data. It has a user-friendly web interface together with genome browser and pathway viewer, which display data in an intuitive manner to the soybean researchers, producers and consumers.

**Conclusions:**

SoyKB addresses the increasing need of the soybean research community to have a one-stop-shop functional and translational omics web resource for information retrieval and analysis in a user-friendly way. SoyKB can be publicly accessed at http://soykb.org/.

## Introduction

A hallmark of modern biology is tremendous amounts of complex omics data, which require large-scale data management, comprehensive computational analyses, fast retrieval and efficient integration for better understanding of the data and more effective hypothesis generation. Such an infrastructure has already been developed for some model organisms such as TAIR [1] for *A. thaliana*, Wormbase [2] for *C. elegans*, MGD [3] for *M. musculus*, SGD [4] for *S. cerevisiae*, Flybase [5] for *D. melanogaster*, Oryzabase for *O. sativa *[6] and Gramenefor grasses [7]. There are a number of soybean-specific databases such as Phytozome [8], Soybase [9], Soybean Genome database [10] and Soybean Genomics and Microarray Database [11]. However, these databases do not contain comprehensive large-scale data for soybean. Since the newly sequenced *G. max* genome became available in 2010 [12], the focus of soybean research has shifted towards performing genome-scale experiments, leading to a deluge of biological data being generated. There is an overwhelming amount of high-throughput data including transcriptomics, proteomics and metabolomics data, generated by labs working on soybean. These data can benefit the entire research community as well as soybean producers, consumers and breeders if compiled, integrated and utilized in a novel and comprehensive way.

Motivated by this emerging need that cannot be addressed by existing soybean databases, we conceptualized and developed Soybean Knowledge Base (SoyKB). SoyKB was designed in a modular fashion with easily expandable architecture, making it feasible to accommodate any new additional requirements for years to come. It seamlessly integrates the biological data for genes/proteins, microRNAs (miRNAs), metabolites and SNPs using a unified framework of genome visualization, biological function annotation, and pathway information. It provides users with a web portal to bring their data into SoyKB and compare with the huge inventory of public data in SoyKB. Many of SoyKB entries are also linked to other soybean databases such as Soybase [9] to allow easy and seamless navigation between the two.

## Database structure, design and implementation

**Figure 1:**
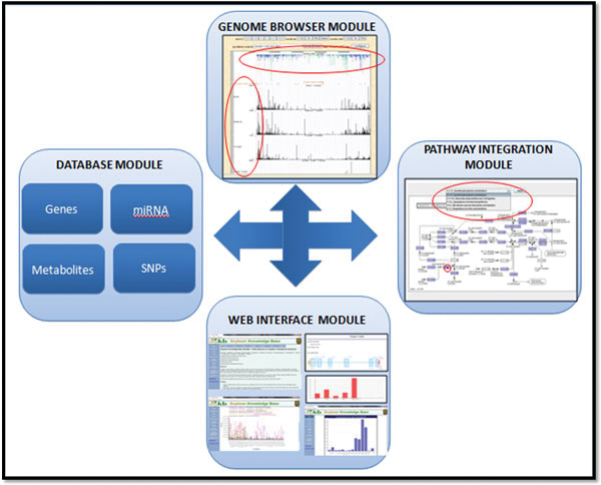
**SoyKB architecture.** SoyKB architecture showing the database, web interface, genome browser and pathway integration modules.

SoyKB is maintained on a Linux server equipped with 8 CPU and 16 GB memory, which hosts both the database and the web interface. It has a modular architecture (as shown in Figure 1) consisting of four modules.

### 1. MySQL database module

The site uses a MySQL database to store large amounts of experimental data and their annotations or analysis results. Through various fast search capacities and tools in SoyKB, the search result is presented in an organized manner that allows for further analysis. This database module incorporates and integrates all the soybean genomics and experimental omics data from various experiments. It is designed to contain information on the four entities namely genes/proteins, miRNAs, metabolites and SNPs.

### 2. Web interface module

SoyKB runs on an Apache [13] server, and was built using PHP [14] for its server-side code. Using standard HTML, CSS, and JavaScript for the client-side presentation, SoyKB was professionally designed to be user-friendly and appealing. The website is designed to provide access to the stored information through a web interface, where researchers and soybean producers can search and retrieve information about whole genome, access data from different experimental conditions and integrate relevant information into specific pathways. Special attention has been paid to the security and permissions of the site. SoyKB has four tiers of registration, which control different levels of access to the public and private data. It allows users of certain levels to share their expertise by adding comments to the data. It also provides links to interesting nutritional facts about soybeans to build connections among soybean researchers, producers and consumers.

### 3. Genome browser module

All the genomic data in SoyKB has been deposited into a genome browser, which is set up locally for soybean utilizing the architecture provided by UCSC [15]. The module allows users to visualize the gene models and their supporting evidence, SNPs and other experimental data such as gene expression profiles of RNA-Seq, microarray and small RNA. Users can visualize the entire chromosome in a single view to help understand the overall picture. The browser also allows users to zoom in and out to focus on regions of their interest and gives the users the flexibility to load or hide any experimental tracks.

### 4. Pathway integration module

This module integrates data from various experimental conditions and portrays the information on pathways to highlight expressed genes/proteins and metabolites based on the selected microarray, RNA-Seq, proteomics, and metabolomics data.

## Data sources

The data in SoyKB comes from multiple sources. Many of the data incorporated in SoyKB are public data and accessible to all users without login. Currently, SoyKB contains information about 75,778 gene entities, 129 miRNAs and 959 annotated metabolites for Williams 82 cultivar of *G. max*. It also has information regarding 7947 SNPs between cultivars Williams 82 and Forrest as well as 2631 SNPs between Magellan and PI567516C. The gene models, genomic sequences and functional annotation information were acquired from Gmax 1.0 release [12] of soybean genome from Phytozome. The gene models contain sequence-based evidence from EST, 5' RATE (Robust analysis of 5'-transcript ends) and full-length cDNA experiments.

SoyKB has many microarray experimental datasets for public access under 99 stress conditions and 25 tissue types acquired from NCBI GEO [16] and Array Express [17], in addition to 7 leaf and root tissue types and time-course data generated by our collaborators, currently only available for private access. The data for private access (as requested by our collaborators or the submitters) are password protected until ready for public access. The repository also has experimental data for 28 Illumina RNA-Seq experiments covering various tissue types and time points, all available for public access. Proteomics datasets are publicly available for seeds, roots and roothairs for multiple time points, conditions and replications. The metabolomics datasets came from the SoyMetDB database [18] and have been fully incorporated in SoyKB.

SoyKB also hosts data regarding 129 miRNAs and their expression abundances from five small RNA tissue libraries including root, nodule, flower, seed and stripped root [19]. It also has a set of 7947 SNPs [20] and another set of 2631 SNPs (Nguyen lab, unpublished) available for public and private access, respectively. The pathway information was acquired from KEGG [21], Genebins [22] and Mapman [23].

## Access and retrieval

**Figure 2:**
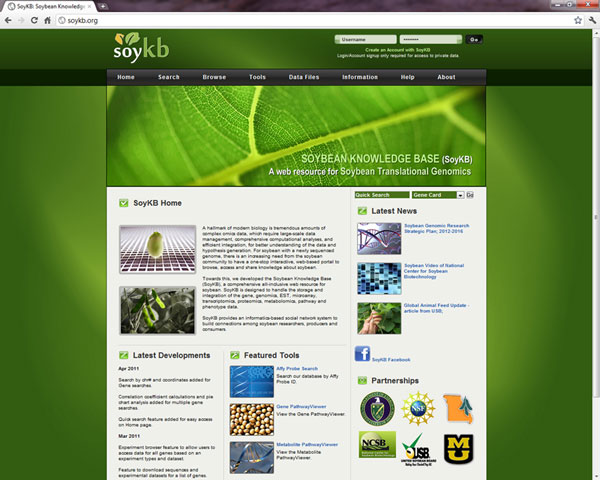
**SoyKB homepage.** SoyKB homepage http://soykb.org/ shows the menu bar for navigation, login, quick search tab and highlight of the developments.

The SoyKB home page as shown in Figure 2 provides users with several entry points to access the vast amount of information stored in it. Users can choose to navigate through the website by clicking on any of the menus highlighted on the top menu bar or simply using the quick search tab on the homepage.

### 1. Website browsing

Each entity in SoyKB has a dedicated entity card page containing all information associated with that entity in the database.

#### i. Gene card

**Figure 3:**
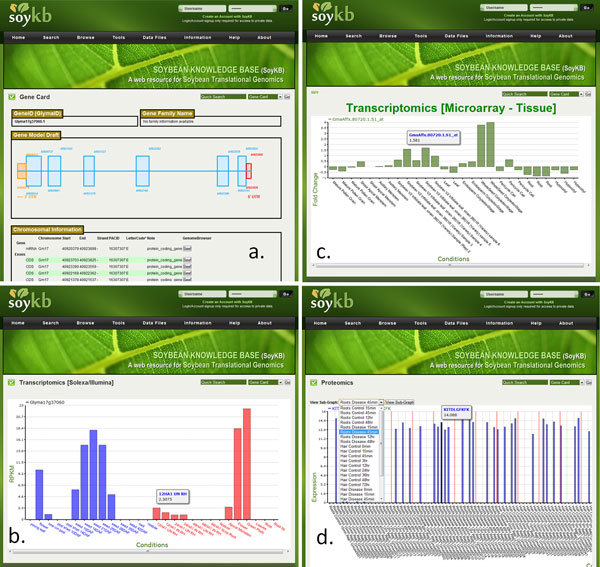
**An example of gene card page. **Gene card pages for gene Glyma17g37060.1 showing the following: (a) gene model and chromosomal coordinates; (b) RNA-Seqtranscriptomics expression profiles; (c) microarray transcriptomics expression profiles; (d) proteomics expression profiles.

**Figure 4:**
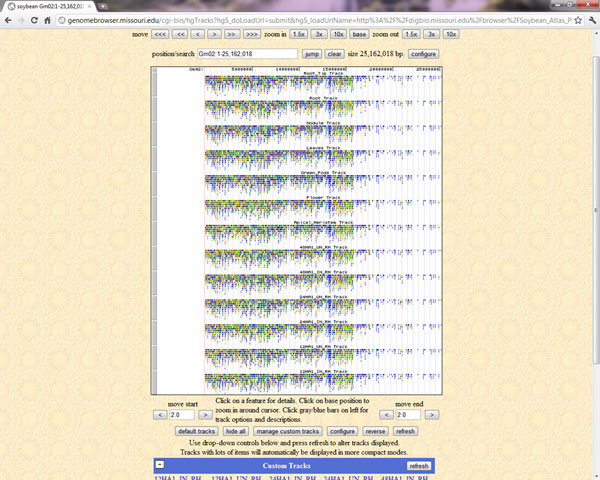
**Genome browser.** An example of genome browser showing a region of chromosome 2 and its expression profiles from RNA-Seqtranscriptomics datasets.

The gene card page shows information about the gene name, gene family information including transcription factors, its gene model with the exon, intron, and UTRs (untranslated regions); links to genome browser, chromosomal coordinates with codes for supporting evidence, cDNA, CDS (coding sequence), and protein sequences, functional annotations including domain information from Pfam, Panther, and KOG; and links to pathway viewer and 3D protein structure viewer as shown in Figure 3a. It also provides links to the alternative gene models if predicted and lists any overlapping SNPs between Williams 82 and Forrest genotypes that fall within the gene coordinates. The gene card page provides access to the sequence based (EST, 5' RATE and full-length cDNA) experimental data and transcriptomics data from microarray and Solexa/Illumina RNA-Seq experiments in addition to other proteomics datasets (Figure 3b,c). Users can apply the sub-graph viewing feature to select a specific experimental condition and only focus on replicates for that particular condition while hiding the remaining data points. The genome browser is linked on each gene card page and can be used to visualize the experimental data for that gene on the browser (Figure 4). SoyKB also provides links to the dynamically conducted literature search result for the gene/protein in the gene card page under "References".

#### ii. miRNA card

The miRNA card stores information about the experimental or predicted miRNAs, mature miRNA sequence, miRNA family, links to corresponding miRBase accession ID and family, expression abundance in small RNA libraries, and predicted target genes.

#### iii. Metabolite card

The metabolite card page provides users with information about metabolites including alias names, mass-to-charge ratios, retention times, chemical formula, chemical structure, molecular weight, links to the pathway viewer and Simplified Molecular Input Line Entry Specification (SMILES) formula. It also provides expression data from GCMS-polar, GCMS-nonpolar and LCMS datasets plotted as bar graphs for easy visualization.

#### iv. SNP card

The SNP card includes information about the predicted SNPs, their chromosomal positions,reference bases, consensus bases, read quality, and sequencing depth along with other quality scores. It also lists any genes where the SNP overlaps and falls within a gene model's coordinates.

### 2. Querying the database

**Figure 5:**
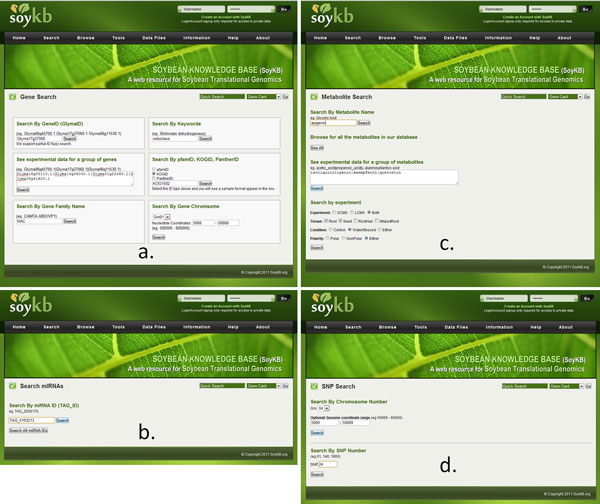
**Querying the database for gene, miRNA, metabolite and SNP entities.** SoyKB has various options for querying (a) gene; (b) miRNA; (c) metabolite and (d) SNP.

The data in SoyKB can be queried is multiple ways. Searches can be made based on a single gene, miRNA, metabolite or SNP using the "Search" menu on the top bar or the "Quick Search" tabonthe homepage. All queries support partial fuzzy search without an exact or complete keyword match. For the genes, entity search can be made using partial or complete gene names, keywords, domain IDs from Pfam/Panther/KOG, gene family names or by specifying a location using nucleotide coordinates on a particular chromosome as shown in Figure 5a. The miRNA entity can be searched using the miRNA ID or simply by clicking on the "Search all miRNA IDs" tab to obtain all miRNAs in the database (Figure 5b). Metabolite entities in the database can be accessed via a metabolite keyword search or by utilizing the link to browse all metabolites in the database. Users can also search metabolites based on a combination of experiment types, tissue types, experimental conditions and polarity to narrow the search down to specific lists of metabolites as in Figure 5c. The SNPs data can be accessed using SNP number or selecting a genomic location using a range of nucleotide positions and a chromosome number (Figure 5d). SoyKB also allows users to query multiple genes or metabolites in a single query and combine the search results.

### 3. Bulk downloads

Users can download data for their gene lists of interest by using the download capacity on SoyKB. The chromosome coordinates for genes, exons and UTR; CDS, cDNA and protein sequences; Pfam, Panther and KOG domains; and microarray, transcriptomics, proteomics, EST, 5'RATE and full-length cDNA are some of the data currently available for bulk download.

### 4. Data submission

To expand the data repository, SoyKB also provides interested users the capacity to contribute their data to SoyKB and choose when to allow that data for public access. This can be done using the "Upload Data File" option under the "Data Files" menu on the top menu bar. Based on the type of data chosen for submission, the selected option will specify the accepted formats for each data type. Accepted file types include .txt, .xls, .xlsx and .csv. Data submissions undergo internal evaluations to look for inconsistency in the data format and any missing or unreliable information, before getting uploaded to the database.

## Useful tools

SoyKB contains Java applications for displaying a pathway with genes and metabolites as well as Flash-powered experimental data charts and 3D protein structures. SoyKB also provides users with an array of comprehensive analysis tools including co-expression analysis for multiple genes/metabolites, Affymetrix probe ID mapper, and gene family browser.

### 1. Pathway viewer

The pathway viewer is targeted towards integrating data from various experimental conditions and portraying the data on pathways to highlight expressed genes and metabolites based on the selected data. It runs as a Java application that uses standard Apache web server technologies such as PHP version 5 to manage web-based data input and query.

**Figure 6:**
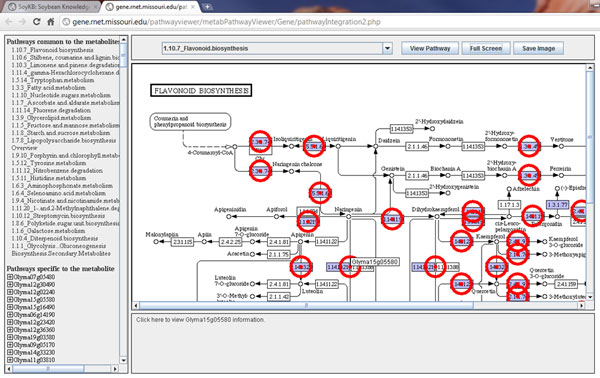
**Pathway viewer.** Multiple genes of interest in the flavonoid biosynthesis pathway are viewed simultaneously shown by highlighted circles. The left panel shows a list of other pathways containing any genes in the list.

Annotated genes and metabolites are mapped to the pathways by combining data from KEGG [21], Genebins [22] and Mapman [23]. This is achieved by integrating the mapping files of annotated genes, compounds, reactions and enzymes with the pathway XML and image files from the KEGG, Genebins and Mapman. Users can enter a single gene/metabolite or a list of genes and metabolites and the respective pathway viewer tool displays all the identified pathways and highlights the genes/metabolites on the pathway with blinking circles around their positions (Figure 6). When queried with multiple genes or metabolites, the tool also lists the most commonly seen pathways for the entered list, which is useful for users to identify most represented pathways in their list of interest (Figure 6). The metabolite pathways are further divided into metabolic and non-metabolic pathways for simplicity. Clicking on the highlighted gene/metabolite in the pathway shows a link to the respective gene card or metabolite card page in the bottom panel for easy access.

### 2. Protein 3D structures

**Figure 7:**
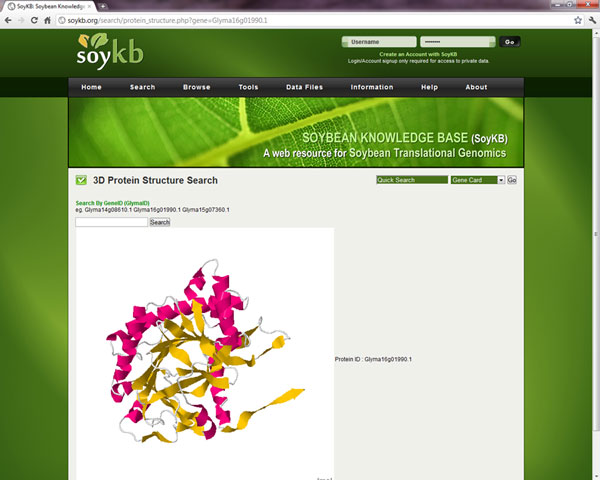
**Protein 3D structure viewer.** Visualization of predicted protein 3D structure for gene Glyma16g01990.1 showing alpha-helices in purple and beta-sheets in yellow.

Protein 3D structural models for all proteins have been predicted using MULTICOM [24] and incorporated on the gene card page using Jmol [25]. Figure 7 shows an example of the protein 3D structure for gene Glyma16g01990.1.

### 3. Co-expression analysis

**Figure 8:**
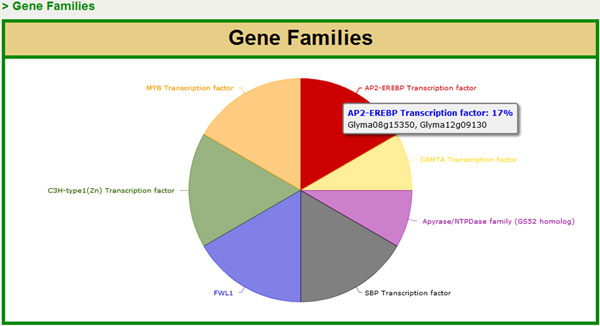
**Pie chart for gene family distribution.** Multiple-gene search showing the gene family distribution for the multiple query genes.

Multiple gene or metabolite searches can be conducted in SoyKB and data can be retrieved for cross comparison among them. For a given set of genes, pie charts can be generated based on the domain annotation or gene family categories as shown in Figure 8.

### 4. Affymetrix probe ID mapper

Many microarray experiments provide expression values for a list of probes instead of genes. We have developed the Affymetrix probe ID mapper tool to allow researchers to map probes to genes automatically using the most up-to-date gene models. The gene lists identified are all linked to the respective gene card pages for easy access to other information about the genes.

### 5. Gene family browser

SoyKB also allows users to browse entire gene families by using the "Browse" feature. "Gene Families" include the transcription factor families predicted in the SoyDB website [26], the cytochrome P450 gene families identified by Guttikonda et al. [27], and a few other gene families. Selecting a gene family provides a list of all genes known to belong to this gene family with individual genes linked to their gene card pages.

### 6. Blast sequence similarity

**Figure 9:**
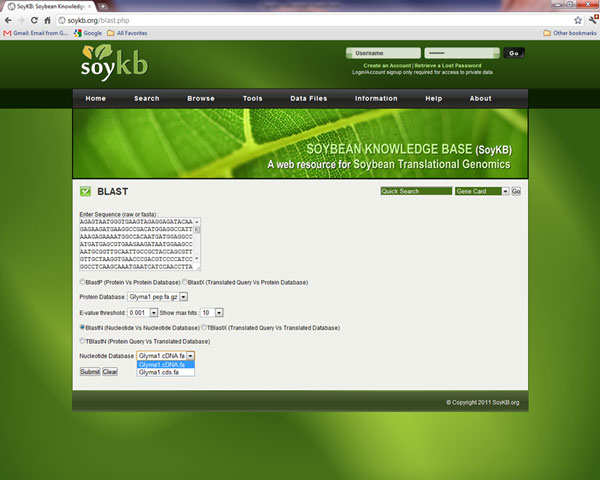
**BlastSequence similarity tool.** Sequence similarity searches against the protein, cDNA, and CDS databases can be conducted using query sequences and corresponding Blast options.

SoyKB also supports sequence similarity searches against the *G. max *protein, cDNA, and CDS databases using protein or nucleotide query sequences (Figure 9). This allows the users to find the closest matches in soybean genome to their sequence of interest.

### 7. Motif prediction and web logo

**Figure 10:**
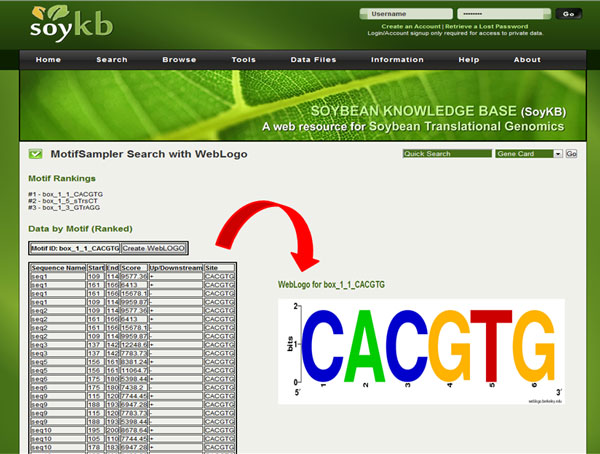
**Motif prediction and web logo.** Conserved motifs predicted in a set of nucleotide sequences and web logo created for the top ranking motif.

The Motif Sampler [28,29] tool allows users to predict common conserved motifs for multiple nucleotide sequences and rank them according to the scores. Users can use this tool to find the conserved motifs in a list of genomic sequences and further create a web logo [30] using the online tool as shown in Figure 10.

## An application example

**Figure 11:**
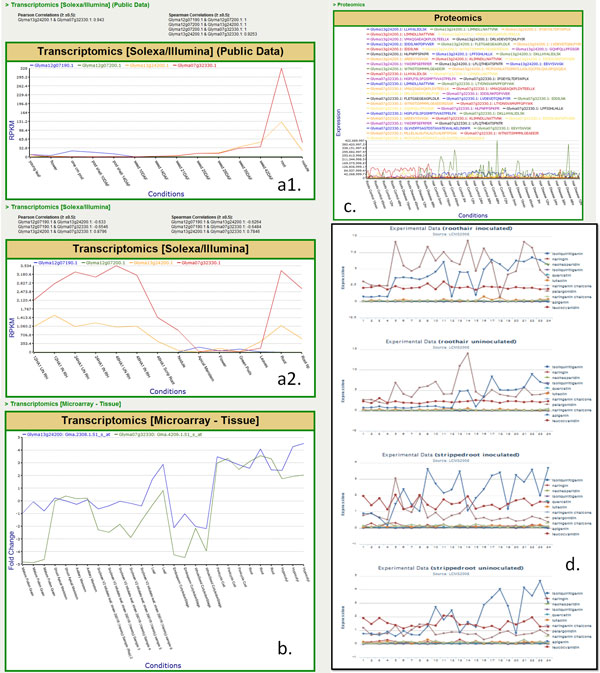
**Cytochrome P450 Omics expression analysis in SoyKB.** Expression analysis for four cytochrome P450 genes showing their (a) RNA-Seqtranscriptomics profiles; (b) microarray transcriptomics profiles; (c) proteomics profiles and (d) metabolomics profiles for all metabolites in the flavonoid biosynthesis pathway.

**Figure 12:**
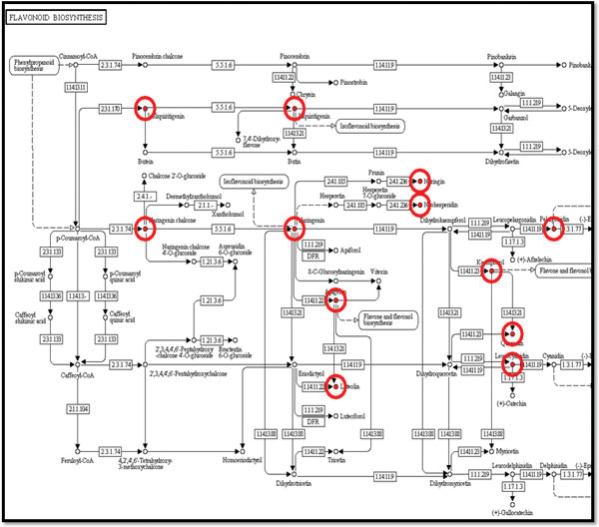
**Metabolites in the flavonoid biosynthesis pathway.** Flavonoid biosynthesis pathway with metabolites highlighted in red circles, indicating additional available data in SoyKB.

Here we provide a case study to show an application of SoyKB by integrating transcriptomics, proteomics and metabolomics data for genes involved in the flavonoid biosynthesis pathway. We studied four cytochrome P450 genes (Glyma12g07190, Glyma12g07200, Glyma13g24200, and Glyma07g32330) to compare their expression patterns across RNA-Seqtranscriptomics, microarray gene expression and proteomics datasets in root tissue conditions. RNA-Seqtranscriptomics datasets show that both Glyma13g24200 and Glyma07g32330 have elevated expression in the root tissue (Figure 11a1) as well as in the root tip and root hair tissues (Figure 11a2) with very high Pearson and Spearman correlation coefficients between the two. The same two genes also showed high expression in root tissues in multiple microarray transcriptomics datasets as well (Figure 11b). We also found that the same two genes showed significant expressions in root and root hair conditions in proteomics datasets as shown in Figure 11c. We studied these genes and identified that they werepresent in the flavonoid biosynthesis pathway. We then extracted all the soybean genes in the flavonoid biosynthesis. We further identified all metabolites in SoyKB that are known in the flavonoid biosynthesis pathway as shown in Figure 12. We also studied the metabolites expression patterns and identified 12 metabolites (liquiritigenin, naringin, neohesperidin, isoliquiritigenin, kaempferol, quercetin, luteolin, naringenin, elargonidin, naringeninchalcone, apigenin and leucocyanidin) to be significantly expressed in the root hair and stripped root with inoculated and uninoculated conditions as shown in Figure 11d. All of this data can is already integrated in SoyKB and can be easily retrieved using simple query searches to draw meaningful inferences.

## Future developments

**Figure 13:**
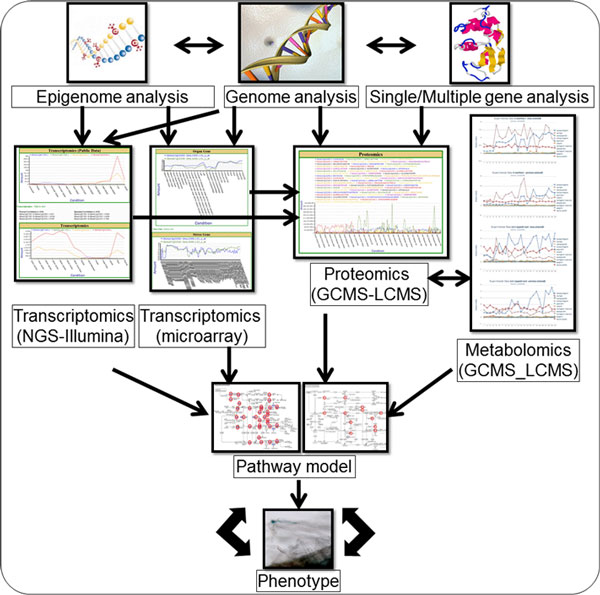
**Integration of different omics data sets in SoyKB.** SoyKB holds data for all types of omics experiments. This figure shows future development for possible crosstalks among these omics datasets.

SoyKB has a unique capability of hosting all kinds of omics data for soybean translational research. It has the infrastructure to integrate different omics datasets and help draw hypotheses and conclusions about phenotypic changes as a result of treatment conditions. Cross comparisons can also be made to evaluate the expression at transcriptomics levels against those at proteomics and metabolomics levels in the same experimental conditions. We will enrich the data analysis and hypothesis generation capacity for various crosstalks among omics datasets as outlined in Figure 13. For example, we are working on methods for data integration and developing an inference engine, which utilizes the transcriptomics, proteomic and metabolomics data from the root hair condition to outline the networks of genes and metabolites of significance in inoculated and un-inoculated experimental data. These tools will become part of SoyKB after sufficient testing and can later be applied to data from any conditions.The tools will also serve as a useful resource for biologist to conduct *in silico *studies on SoyKB directly.

Many other new capacities are also currently under development, including tools to handle epigenomics methylation data, breeder's toolkit with QTL and traits information, comparison against *G. soja*, and tools for phenotype prediction using omics data. The SoyKB development team is actively working towards incorporating more datasets and making them available for public access. We will setup an ftp site to give users access to the entire datasets. We will also provide mirror sites for more stable and fast access of SoyKB around the world.

## Competing interests

The authors declare that they have no competing interests.

## Authors' contributions

TJ is the primary designer and lead developer of SoyKB in addition to managing the development team. TJ also conducted bioinformatics analysis on the datasets hosted in SoyKB and drafted the initial manuscript. KP, MRF, LDF, QY and JRC were involved in database and web interface development. ML, LB, BV and XW performed experiments, generated data and provided feedback. ZW and JC predicted protein structures for all genes. DX provided overall guidance. GS and HTN provided helpful suggestions. All authors read and approved the final manuscript.
